# Clinical Presentation and Outcomes of Myocarditis Among the COVID-19 Pediatric Population: A Review of 100 Cases

**DOI:** 10.7759/cureus.69885

**Published:** 2024-09-21

**Authors:** Ashfaq Ahmed, Asad Iqbal, Amir Abdullah, Muhammad Irshad, Muhmamad Qasim Khan

**Affiliations:** 1 Pediatric Department, Saidu Teaching Hospital, Saidu Sharif, PAK; 2 Pediatric Department, Lady Reading Hospital Peshawar, Peshawar, PAK; 3 Pediatric Department, Bacha Khan Medical College, Mardan, PAK; 4 Pediatric Department, Mardan Medical Complex, Mardan, PAK

**Keywords:** cardiac enzymes, covid-19, ejection fraction, hospital stay, immunoglobulin, myocarditis, outcomes, pediatric clinical presentations, troponin-i

## Abstract

Background: COVID-19 has been associated with myocarditis in the pediatric population, leading to severe cardiac complications.

Objective: To determine the clinical presentations and outcomes of myocarditis among the COVID-19-positive pediatric population.

Materials and methods: This retrospective cross-sectional study included 100 cases from the Saidu Group of Teaching Hospitals, Swat. Inclusion criteria involved children of both genders, confirmed COVID-19 by PCR, and a myocarditis diagnosis. Exclusion criteria were other comorbid conditions, incomplete records, and age over five years. Data included age, gender, weight, clinical features, cardiac enzyme levels, ejection fraction, PCR results, immunoglobulin treatment, outcomes, and hospital stay duration. Statistical analysis was performed in SPSS employing descriptive statistics, chi-square tests, and Fisher's exact tests.

Results: The mean age was 24.72±18.67 months, with 67 males and 33 females. Irritability was noted in 18 children, cyanosis in 27, and cough in 74. Tachycardia was observed in 91 children. Elevated cardiac enzymes and positive Troponin-I levels were found in 91 and 84 children, respectively. The mean ejection fraction was 36.29±9.12%. The average hospital stay was 7.11±2.49 days. Among 100 children, 26 died while 74 recovered. Immunoglobulin administration showed no significant difference between the expired and improved groups (p=0.6). Longer hospital stays were associated with mortality (p=0.002). Troponin-I levels were significantly higher in the expired group (p=0.01).

Conclusion: Key factors associated with poor outcomes include low ejection fraction, elevated cardiac enzymes, positive Troponin-I levels, and shorter hospital stays.

## Introduction

Myocarditis, characterized by inflammation of the heart muscles, is a significant condition that can affect children, leading to severe cardiovascular complications [1]. The etiology of myocarditis in children is diverse, encompassing a range of infectious agents, autoimmune processes, and idiopathic factors [2]. Viral infections are the most common cause, with viruses such as enteroviruses, adenoviruses, and more recently, the novel coronavirus (SARS-CoV-2) being implicated in pediatric myocarditis cases [3].

The clinical presentation of myocarditis in children varies widely, from asymptomatic cases to severe heart failure and cardiogenic shock, complicating early diagnosis and management [4]. Common symptoms include chest pain, fatigue, palpitations, and shortness of breath. Chest pain in pediatric myocarditis is often caused by inflammation of the heart muscles, irritating the pericardium or myocardium [5]. This can lead to sharp discomfort, especially with breathing or lying down. The inflammatory response involves immune cells infiltrating the heart muscle, causing tissue damage and releasing pro-inflammatory cytokines that contribute to pain. Fatigue results from the heart's decreased ability to pump blood efficiently, leading to reduced oxygen delivery to tissues [6]. The inflammation and myocardial damage reduce contractile function, forcing the body to work harder to meet its metabolic needs, leading to generalized fatigue and weakness. Palpitations are caused by arrhythmias due to inflammation disrupting the heart's electrical pathways. These irregular heartbeats can range from benign to severe [7]. Shortness of breath is usually due to heart failure or pulmonary congestion, as the weakened heart cannot pump effectively, leading to pulmonary edema. In severe cases, heart failure signs like tachycardia, hypotension, and edema may occur. Tachycardia compensates for reduced stroke volume due to myocardial damage, maintaining blood flow. Hypotension results from ineffective blood pumping, exacerbated by systemic inflammation. Edema happens due to fluid retention caused by decreased cardiac output, leading to fluid accumulation in tissues [8].

With the advent of the COVID-19 pandemic, there has been an alarming increase in the incidence of myocarditis among pediatric patients. COVID-19-associated myocarditis has emerged as a notable complication, presenting additional challenges to healthcare providers [9]. These cases are often characterized by severe inflammatory responses, sometimes overlapping with multisystem inflammatory syndrome in children (MIS-C), further complicating the clinical picture [10].

This study is the first of its kind in Swat's pediatric population. It addresses a critical gap in the local literature, where data on COVID-19 complications like myocarditis is sparse despite the significant impact of infectious diseases on children. During the COVID-19 pandemic, this complication has become notable in children, who, despite generally having milder symptoms, can suffer severe inflammatory responses. By providing statistics on mortality associated with COVID-19 myocarditis, this study can alert clinicians and administrators to the urgent need for updated skills and tools for quick diagnosis and management, ultimately enhancing local healthcare strategies and improving patient outcomes.

The objective of this study was to determine clinical presentations and outcomes of myocarditis among the COVID-19-positive pediatric population.

## Materials and methods

This retrospective cross-sectional study, comprising 100 cases, was conducted from August 2022 to August 2023 and utilized records available in the Department of Pediatrics at Saidu Group of Teaching Hospitals, Swat, Pakistan. Non-probability consecutive sampling was employed. Inclusion criteria encompassed both genders, confirmed cases of COVID-19 by PCR, and a myocarditis diagnosis. Exclusion criteria involved other comorbid conditions, incomplete records, and age over five years.

The sample size was 92, calculated by OpenEpi software with a 7% margin of error and a 95% confidence level, using a 13.5% mortality rate among children with COVID-19 from a previous study [11]. We included 100 cases to further increase the power of the study.

The diagnosis of myocarditis was confirmed through echocardiography findings of decreased systolic function (evidenced by EF <55%), left ventricular (LV) dysfunction, and cardiomegaly; chest X-ray indicating cardiomegaly; laboratory tests showing elevated cardiac enzymes such as troponin; and clinical presentation characterized by tachycardia, weak pulse, and hypotension.

The recorded data included age in months, gender, weight in kilograms, clinical features, cardiac enzyme levels, ejection fraction, COVID-19 status on PCR (positive/negative), treatment with immunoglobulin, outcome, and duration of hospital stay (in days).

Statistical analysis was conducted using SPSS version 27 (IBM SPSS Statistics, Armonk, NY). Descriptive statistics were calculated as the mean and standard deviation (SD) for continuous variables and as frequencies and percentages for qualitative variables. Outcomes (death or recovery) were compared in relation to immunoglobulin administration, length of hospital stay, ejection fraction, PCR results, antibody presence, and cardiac enzyme levels in children with COVID-19-associated myocarditis. Chi-square tests or Fisher's exact tests (for cell counts <5) were used for these comparisons. A significance level of p <0.05 was considered statistically significant.

Ethical approval was obtained from the Ethical Review Board of Saidu Group of Teaching Hospitals, Swat (No. 119/ERB), and informed consent was obtained from parents for the use of their children's data for research.

## Results

**Figure 1:**
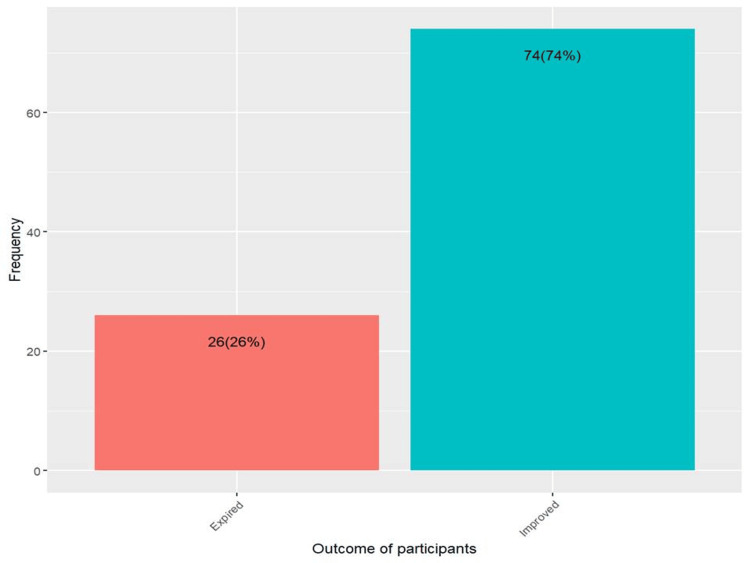
Outcome in children with COVID-19 myocarditis

Fever was present in all participants. The mean age and weight were 24.72±18.67 months and 10.17±3.55 kg, respectively. There were more females (n=67) than males (n=33). In a study of 100 children with COVID-19-associated myocarditis, irritability was observed in 18% of the children (n=18), while 82% (n=82) did not exhibit this symptom. Cyanosis was present in 27% (n=27) of the children, whereas 73% (n=73) did not show signs of cyanosis. Cough was reported in 74% (n=74) of the participants, with 26% (n=26) not experiencing this symptom. A significant majority of the children, 91% (n=91), exhibited tachycardia compared to 9% (n=9) who did not. Elevated cardiac enzymes were found in 91% (n=91) of the cases, while 9% (n=9) had normal levels. Troponin-I levels were positive in 84% (n=84) of the children, and negative in 16% (n=16). The mean ejection fraction was 36.29% with a SD of 9.12%. PCR tests were positive in 36% (n=36) of the children, with 64% (n=64) testing negative. Antibodies were detected in 48% (n=48) of the participants, while 52% (n=52) did not have detectable antibodies. Immunoglobulin was administered during treatment to 35% (n=35) of the children, and 65% (n=65) did not receive it. The average hospital stay was 7.11 days with an SD of 2.49 days (Table 1). Of the 100 total participants, 26 children died, while 74 recovered (Figure 1).

**Table 1:** Distribution of laboratory and clinical features of COVID-19-associated myocarditis children

Variable	Characteristic	N=100
Irritability	No	82 (82.00)
	Yes	18 (18.00)
Cyanosis	No	73 (73.00)
	Yes	27 (27.00)
Cough	No	26 (26.00)
	Yes	74 (74.00)
Tachycardia	No	9 (9.00)
	Yes	91 (91.00)
Cardiac enzymes	Negative	9 (9.00)
	Raised	91 (91.00)
Troponin-I	Negative	16 (16.00)
	Positive	84 (84.00)
Ejection fraction	Mean±SD	36.29±9.12
PCR	Negative	64 (64.00)
	Positive	36 (36.00)
Antibodies	Negative	52 (52.00)
	Positive	48 (48.00)
Immunoglobulin given during treatment	No	65 (65.00)
	Yes	35 (35.00)
Stay in hospital (days)	Mean±SD	7.11±2.49

In this study comparing outcomes in children with COVID-19-associated myocarditis, 26 children expired while 74 improved. Immunoglobulin was administered during treatment to 30.77% (n=8) of the expired group and 36.49% (n=27) of the improved group, with no significant difference (p=0.6). Hospital stays longer than one week were significantly less common in the expired group (7.69%, n=2) compared to the improved group (40.54%, n=30) (p=0.002). All children who expired had low ejection fractions (100%, n=26) compared to 95.95% (n=71) in the improved group, though this was not statistically significant (p=0.6). PCR results were positive in 50% (n=13) of the expired group and 31.08% (n=23) of the improved group (p=0.084). Antibodies for COVID-19 were positive in 65.38% (n=17) of the expired group and 41.89% (n=31) of the improved group, showing a significant difference (p=0.039). Raised cardiac enzymes were observed in 96.15% (n=25) of the expired group and 89.19% (n=66) of the improved group (p=0.4). Troponin-I levels were positive in all expired cases (100%, n=26) compared to 78.38% (n=58) in the improved group, which was statistically significant (p=0.01) (Table 2).

**Table 2:** Comparison of outcome with respect to immunoglobulin given, stay in hospital, ejection fracture, PCR, antibodies, and cardiac enzymes in COVID-19-associated myocarditis children *P-value <0.05 is significant

Characteristic	Outcome		P-value^*^
	Expired, N=26	Improved, N=74	
Immunoglobulin given during treatment			0.6
No	18 (69.23)	47 (63.51)	
Yes	8 (30.77)	27 (36.49)	
Stay in hospital			0.002
More than 1 week	2 (7.69)	30 (40.54)	
Up to 1 week	24 (92.31)	44 (59.46)	
Ejection fraction			0.6
Low	26 (100.00)	71 (95.95)	
Normal	0 (0.00)	3 (4.05)	
PCR for COVID-19			0.084
Negative	13 (50.00)	51 (68.92)	
Positive	13 (50.00)	23 (31.08)	
Antibodies for COVID-19			0.039
Negative	9 (34.62)	43 (58.11)	
Positive	17 (65.38)	31 (41.89)	
Cardiac enzymes			0.4
Negative	1 (3.85)	8 (10.81)	
Raised	25 (96.15)	66 (89.19)	
Troponin-I			0.01
Negative	0 (0.00)	16 (21.62)	
Positive	26 (100.00)	58 (78.38)	

## Discussion

In this study of 100 children with COVID-19-associated myocarditis, fever was a universal symptom, aligning with its common presentation in pediatric COVID-19. Similar results were found in previous studies [12,13]. There were more males (n=67) than females (n=33), suggesting a potential gender predisposition to severe COVID-19 or myocarditis [14].

Irritability was observed in 18 children, which could be due to the systemic inflammation and discomfort associated with myocarditis. Cyanosis was noted in 27 children, indicative of severe hypoxemia likely resulting from impaired cardiac function. Cough was reported in 74 children, reflecting the respiratory involvement typical of COVID-19. Tachycardia was present in 91 children, a key indicator of myocarditis due to inflammation affecting heart function. Elevated cardiac enzymes were found in 91 cases, signaling myocardial injury. Troponin-I levels were positive in most of the children, confirming myocardial damage [15]. A previous study reported similar findings; however, that study was conducted on adults [16]. 

The mean ejection fraction was 36.29%, showing significantly reduced cardiac function. The reduced ejection fraction in children with COVID-19-associated myocarditis is due to a combination of direct viral damage, inflammatory responses, immune-mediated injury, and potential metabolic disturbances. Each of these factors can impair the myocardium's ability to contract and pump blood efficiently, leading to decreased cardiac output and reduced ejection fraction [17].

Immunoglobulin was administered to 35 children in an attempt to modulate the immune response. A systematic review of 14 case reports failed to establish the efficacy of immunoglobulin to reduce mortality among children with COVID-19-associated myocarditis [18].

Of the 100 total participants, 26 children died, while 74 recovered. Immunoglobulin was given to eight of the children who expired and 27 of those who improved. This lack of significant difference suggests that immunoglobulin therapy may not have a decisive impact on survival in this cohort. Similar results were reported in a systematic review [18]. Hospital stays longer than one week were observed in only two of the children who expired, compared to 30 of those who improved. This significant difference indicates that longer hospital stays might be associated with better management and recovery [19].

All 26 children who died had low ejection fractions, compared to 71 out of the 74 children who improved. Although this difference was not statistically significant, it highlights severe cardiac dysfunction as a critical factor in mortality [20].

Raised cardiac enzymes were seen in 25 of the children who expired and 66 of those who improved, underscoring the prevalence of myocardial injury. Troponin-I levels were positive in all 26 children who died, compared to 58 of the children who improved, reinforcing the association between myocardial damage and mortality. Previous studies have shown that Troponin-I levels can be a predictor of mortality among children with myocarditis [21,22].

Limitations of study

A notable strength of this study is its focus on a specific and vulnerable pediatric population, providing valuable insights into COVID-19-associated myocarditis in children under five years old. However, this study has several limitations. As a retrospective cross-sectional study, it is inherently subject to selection bias and may not fully capture the dynamic nature of disease progression. The reliance on existing medical records introduces the potential for incomplete or inaccurate data. The exclusion of children over five years and those with comorbid conditions limits the generalizability of the findings. The study's single-center design may limit the applicability of the results to other settings or populations.

## Conclusions

It was indicated that all participants had fever, and many exhibited additional symptoms such as irritability, cyanosis, cough, and tachycardia. Common findings included elevated cardiac enzymes and positive Troponin-I levels. The average hospital stay was about a week. Out of 100 children, 26 died while 74 recovered. Poor outcomes were associated with factors such as low ejection fraction, elevated cardiac enzymes, positive Troponin-I levels, and shorter hospital stays. Although immunoglobulin administration was part of the treatment for some children, it did not significantly impact the outcomes.
